# Case Management Programs for Improving Integrated Care for Frequent Users of Healthcare Services: An Implementation Analysis

**DOI:** 10.5334/ijic.5652

**Published:** 2022-02-11

**Authors:** Catherine Hudon, Maud-Christine Chouinard, Mathieu Bisson, Astrid Brousselle, Mireille Lambert, Alya Danish, Charo Rodriguez, Véronique Sabourin

**Affiliations:** 1 Département de médecine de famille et de médecine d’urgence, Université de Sherbrooke, 3001, 12e Avenue Nord, Sherbrooke, QC, Canada; 2 Centre de recherche du CHUS, 12e Avenue Nord Porte 6, Sherbrooke, QC, Canada; 3 Faculté des sciences infirmières, Université de Montréal, Pavillon Marguerite-d’Youville, 2375 Chemin de la Côte-Sainte-Catherine, Montreal, QC, Canada; 4 School of Public Administration, University of Victoria, Public Administration, HSD building, Room A302, Victoria, BC, Canada; 5 Centre intégré universitaire de santé et services sociaux du Saguenay–Lac-Saint-Jean, 930 rue Jacques-Cartier E, Chicoutimi, QC, Canada; 6 Department of Family Medicine, McGill University, 5858, Chemin de la Côte-des-Neiges, Montreal, QC, Canada; 7 Patient partner, CA

**Keywords:** implementation analysis, case management program, multiple embedded case study, high users of healthcare services, integrated care

## Abstract

**Introduction:**

Case management programs (CMP) for frequent users of healthcare services presenting complex healthcare needs constitute an effective strategy to improve patient experience of integrated care and to decrease healthcare overuse and cost. This study sought to identify characteristics of these programs, and their implementation contexts, that help to improve patient self-management, experience of integrated care, and healthcare services use.

**Methods:**

A mixed methods multiple embedded case study design was conducted, with six CMP implemented in six hospitals of a region of Quebec (Canada).

**Results:**

Within-case analysis describes the structural, environmental, organizational, practitioner, patient, and innovation level characteristics of each CMP and their services integration outcomes based on patient experience, self-management and healthcare services use. Cross-case analysis suggests that the skills, leadership and experience of the case manager, providers’ access to the individualized services plan, consideration of the needs of the patient and family members, their participation in decision-making, and the self-management approach, impact integrated care and healthcare services use.

**Conclusion and discussion:**

This study underscores the necessity of an experienced, knowledgeable and well-trained case manager with interpersonal skills to optimize CMP implementation such that patients are more proactive in their care and their outcomes improve.

## Introduction

Individuals with chronic conditions sometimes have complex healthcare needs, due to mental health comorbidities and/or social vulnerabilities [1] and become frequent users of healthcare services [2,3,4]. Organizing services to improve care for these patients with complex needs is a priority for healthcare systems [4] and requires an integration of clinical services offered by health and social care professionals, as well as community-based ones [5]. Models of integrated care such as case management [6] improve the quality of care, patient satisfaction, access to care, and care transitions [7,8], and reduce the probability of hospitalization, when compared with usual care [9].

Case management programs (CMP) for frequent users of healthcare services with complex needs constitute an effective strategy to improve patient experience of integrated care and to decrease healthcare overuse and cost [10,11,12]. Case management is a dynamic, systematic and collaborative approach used to ensure, coordinate, and integrate care and services for a clientele. The case manager is a key practitioner or navigator (often a nurse or a social worker) who evaluates, plans, implements, coordinates, and prioritizes services based on individuals’ needs, and offers self-management support in close collaboration with health, social and community partners [13].

A majority of studies have reported the effectiveness of CMP with indicators such as improvement in patient satisfaction and quality of life, and reduction of healthcare services use, ED visits, hospitalization rates, and cost [10,11,12]. Although the evidence in support of CMP is strong, there remains a paucity of evidence about the implementation process that lead to these outcomes in local contexts [14]. The aim of this study was to identify characteristics of CMP, and the contexts where they are implemented, that help to improve patient self-management, experience of integrated care, and healthcare services use.

## Methodology

### Design of the study

This was a case study, more specifically a multiple embedded case study with a mixed-methods design [15]. Such a methodology appears the most appropriate for an implementation analysis in a complex system, and to study cases, with varied contexts, as they evolve over time [15,16]. In addition to allowing for an in-depth analysis of each case, the analysis strategies used in this design help to systematically compare trends observed between cases. It is recommended that four to ten cases be considered [17] in the multiple case study logic of theoretical replication [15]. This study included six cases, where each case was the CMP implemented in each hospital. The three different units of analysis that were interwoven to obtain an in depth understanding of each case were: 1) the hospital (organizational ‘macro’ level); 2) the CMP itself for frequent users of services (‘meso’ level) and 3) the individual (‘micro’ level), more particularly patients who are frequent users.

### Context of the study

The study was realized in the Saguenay-Lac-Saint-Jean region of Quebec, a province in Canada. This region is the third largest territory in Quebec and has a very low average population density of 2.9 inhabitants per square kilometre. Much of the population is French-speaking and less than 1% are immigrants. Compared to the whole Quebec population, the residents of the Saguenay-Lac-Saint-Jean region have lower educational attainment and experience more mental health conditions [18]. In the province of Quebec, regions are divided into administrative sectors referred to as County Regional Municipalities (CRM). In the Saguenay subregion, one of these CRM is served by three hospitals, and in the Lac-Saint-Jean subregion, three of these CRM are each served by a hospital.

### Case management program

In 2008, the Saguenay-Lac-Saint-Jean health and social services agency mandated the six hospitals of its territory to implement CMP for frequent users of healthcare services. Between 2009 and 2015, six CMP, the cases included in this study, were deployed by stakeholders’ committees made up of a coordinator, managers, services coordinators and case managers. CMP aimed to improve self-management support and integrated care, and decrease ED use, hospitalizations as well as healthcare cost. Case managers (a nurse or a social worker or both in dyad) in each of the six hospitals were recruited and trained to the case management approach. The training enabled the case managers to identify patients with complex care needs, assess their specific needs, and develop the individualized service plans (ISP) to respond to those needs in collaboration with the patient, their relatives and other actors involved in the implementation of the ISP, including nurses, social workers, family physicians, pharmacists, and representatives of community organizations.

In 2015, during the data collection of the study, the government of Quebec reorganized the healthcare system by merging local hospitals into larger regional entities in order to centralize health and social services. This resulted in an effort from a single CMP committee made up of a coordinator, a manager, a performance improvement consultant and case managers to standardize the program offered by the six hospitals. Members of the committee also discussed challenges and facilitators to the implementation of the CMP in this new context, as well as factors at the healthcare system level that could influence the case managers’ work. Criteria for enrolment were standardized, targeting patients with more than six ED visits or three hospitalizations in the previous year. Frequent users were identified electronically through hospital admissions and ED records. The provincial healthcare system reorganization had major impacts on clinical, professional, administrative, management and governance aspects of the healthcare system. For example, there was staff turnover at the case manager and manager level, which affected the implantation of CMP in many cases.

### Conceptual framework

Two conceptual frameworks guided this study. First, given that we were interested in the implementation of CMP, we used Chaudoir et al. [19] which proposes five broad categories of factors to consider when evaluating the implementation of an innovation, namely: 1) structural and environmental-level factors; 2) organizational-level factors; 3) practitioner-level factors; 4) patient–level factors, and 5) innovation-level factors. Second, to examine patient experience of integrated care, the model proposed by the National Collaboration in Integrated Care and Support was used [20]. It consists of six dimensions of care integration based on patient experience: 1) consideration of patient and family needs, 2) communication with the patient and between practitioners, 3) access to information, 4) patient involvement in decision-making, 5) care planning, and 6) transitions between various professionals.

### Participants

Key informants involved in the six CMP and healthcare services used by patients with complex health needs were recruited through purposeful sampling [21] in each hospital. Patients recruited were frequent users of hospital services, who had six visits to the ED or more, or three hospitalizations or more in the previous year.

### Data collection

An implementation analysis strategy [22] guided the three methods of qualitative data collection and the method of quantitative data collection. While qualitative methods were used to inform self-management and patient experience of integrated care, quantitative data collection methods allowed the measurement of ED services use.

#### Qualitative data

##### Individual interviews and focus groups

Semi-structured individual interviews (n = 56) and focus groups (n = 11) were conducted between December 2014 and May 2018 with 24 patients, 12 case managers and intermediate managers, 8 senior managers, 12 family physicians, 25 community stakeholders and 6 pharmacists, with interview guides, adapted for each type of actor, and addressed the five main categories of factors of the Chaudoir et al. framework of innovation implementation [19], and the six dimensions of patient experience [20]. Data saturation was not the goal for each group, but the diversity of actors engaged provided a complete representation of each case [23]. All individual interviews and focus groups were audio recorded and transcribed verbatim.

##### Participant observation

A member of the research team performed participant observation during one case management training session, individual case manager activities (n = 6) (e.g. evaluation of targeted patient needs, contacts with patients and their healthcare providers, ISP meetings), and quarterly meetings of the CMP committee of each of the six hospitals (n = 11). The member of the research team was invited to attend all committee meetings and share updates about the research project. These meetings were also an opportunity to consult committee members on how the research project could provide new knowledge that would help them. Data were collected using field notes [21].

##### Document analysis

Minutes of the CMP committee meetings were collected as they provided insight into the characteristics of the CMP and the CMP implementation, including challenges and means to overcome them [24].

#### Quantitative data

##### Clinical and administrative data

Using the hospitals’ Magic Chronique computer software [25], the number of frequent users of ED was recorded monthly for each hospital beginning in December 2012 (the year preceding the start date of the study) and ending on May 2018. Data quality was controlled using an integrated model of information quality and a series of validation algorithms.

### Analysis

#### Qualitative data

For each case, all qualitative data were analysed together as one data corpus using a deductive (themes based on the conceptual frameworks [19,20] and inductive (themes emerging from the data) thematic analysis [26]. All data sources were examined to identify characteristics of CMP, and their contexts that can be related (positively or negatively) to the examined outcomes, i.e. patient self-management, experience of integrated care, and healthcare services use. Qualitative data were managed by two authors who used NVivo V.11 server software (QSR International Pty). Other authors, including an experienced patient partner, participated in the analysis. Persistent observation, and methodological and investigator triangulation were used to ensure credibility [27].

##### Quantitative data

The number of ED frequent users (six visits or more in the previous year) was tabulated for each hospital and represented in one graph to allow for visual comparison.

##### Integration of qualitative and quantitative data

Qualitative and quantitative results were compared for each case [28]. Qualitative data was analysed first, quantitative data second, then cross-analyses merged the two corpora of data [15]. A case history was written for each case (***Table 2***) to summarize merged data [26]. To compare the six case records, three analytic techniques used in case study research were used, namely pattern comparison, search for competing explanations and construction of explanations [15]. Management, data reduction and cross case comparisons were conducted with NVivo V.11 software using matrix queries.

The study was approved by the ethics committee of the Centre for integrated health and social services of Saguenay-Lac-Saint-Jean (2014–015).

## Results

***Table 1*** provides the descriptive characteristics of each of the six CMP.

**Table 1:** Characteristics of the six case management programs.


CHARACTERISTIC	CASE					

	A	B	C	D	E	F

Population of hospital service zone in 2017 (n)	78 824	67 264	22 554	52 855	25 615	31 500

Area of the hospital service zone (km^2^) [29]						

CRM	1 126			2 781	36 770	17 799

City-center	156	216	262	196	296	153

Population density (resident/km^2^)						

CRM	0.6			18.6	0.7	1.8

Main city	384.4	253.9	75.9	237.2	50.3	71.3

Year of CMP creation	2009	2012	2013	2012	2015	2013

Case manager	Nurse-social worker dyad	Social worker	Nurse	First: nurse-social worker dyad Later: only a nurse	Social worker	First: nurse-social worker dyad Later: only a nurse

ISP access modality for healthcare providers (other than case manager)	No access	Hard copy folder (n = 1) in the ED	Hard copy (n = 1) in the ED	Digital folders (n = 3)	Digital (n = 1) folder in the ED	Digital (n = 1) and hard copy (n = 1) folders in the ED


CMP: case management program; CRM: County regional municipality; ED: Emergency department; ISP: Individual service plan.

**Figure 1:**
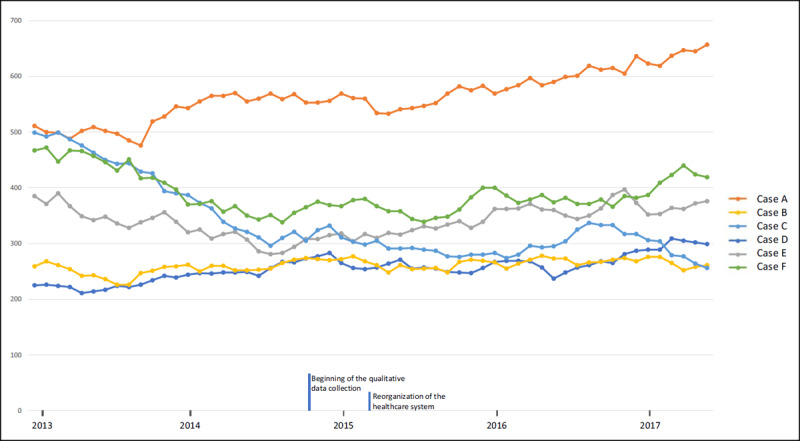
Number of ED frequent users* for each hospital. * FU: 6 ED visits or more in the previous year.

***Figure 1*** illustrates the evolution of the number of ED frequent users during the implementation of the CMP. While the number of frequent users increased considerably in the case A and increased slightly in the case D, an important decrease was observed for the case C and a slight decrease in the case F. These last two cases are considered “success stories”. The cases B and E remain relatively stable.

### Intra-case results

***Table 2*** merged qualitative and quantitative data to present case stories.

**Table 2:** Case management program (CMP) implementation in each setting: case stories.


CASE	DESCRIPTION

**A**	Located in the largest urban centre of the region, this CMP began as a committee of health and social care providers, from various hospital departments, and dedicated to the case management approach with their respective clientele. Then, an official CMP was launched to serve a larger volume of patients. It was led by a nurse-social worker dyad who identified frequent ED users in real-time, thereby ensuring timely patient management. Due to the considerable number of health and social services organizations and providers in this area, interpersonal connections and knowledge between the local actors were difficult to achieve. The care was thus siloed rather than integrated and coordinated. The reorganization of the Quebec healthcare system in 2015 had major impacts on health and social services in general, and on the CMP in particular, including staff turnover at the intermediate and senior management levels and a general climate of insecurity regarding the program sustainability. Due to the reorganization and despite the senior manager’s wish to maintain all CMP activities, the CMP was stopped in November 2015. In May 2018 (the end of the qualitative data collection phase), it had still not been rebooted. The quantitative data indicate a decrease of ED frequent users at the beginning of the program, while a continuous increase in the number of ED frequent users began when the CMP stopped.

**B**	Based in the second largest urban hospital in the region, the CMP served a population with a high prevalence of mental health disorders and social problems. The case manager was a social worker who was not only responsible for coordinating patient care, but who also worked with patients to reduce their high use of care services. Over the course of this study, there was a high turnover of case managers and this seemed to influence the engagement and openness of patients and providers towards the CMP. Moreover, changing leadership among the case managers resulted in mixed program effects over the years. The reorganization of the healthcare system generated staff turnover and shortage as well as lack of other resources (e.g. physical place to work). Consequently, providers were dissatisfied with the CMP implementation and, ultimately, became reluctant to accept any new initiative from senior managers or health authorities, including the CMP. However, with the support of senior managers, the CMP was maintained. The number of ED frequent users remained stable over the study duration.

**C**	The CMP was in a small rural area close to an urban centre; thus, benefiting from geographic proximity between patients and providers. Given that the case manager worked near the family physicians in the clinics, information circulated fluidly, and patient follow up was rapid. Many patients of this CMP were elderly or had chronic pulmonary disease. The case manager was an experienced nurse who was well known by patients and providers before the CMP implementation because this person had been working in the area for many years. Moreover, since becoming the case manager of this CMP in 2013 when it began, this person had worked hard to make herself even more known among the CMP stakeholders and to build relationships with providers. Given these relationships of trust, the initial resistance to the CMP was reduced. Additionally, because the case manager met with patients and ED personnel frequently, follow up was more rapid and efficient. Finally, due to the strong support from senior manager, the case manager had a lot of autonomy, and was thus able to adapt to the needs of patients and providers. Over the course of the case study, the CMP did not change very much. In fact, the potential negative effects of the reorganization of the Quebec healthcare system on the CMP were mitigated by the stability of the case manager and the management team. The number of ED frequent users decreased steadily during the study period.

**D**	The hospital deploying this CMP serves a small territory facilitating inter-professional and inter-organizational collaboration, particularly between family physicians and the case manager. At the beginning of the program in 2012, an emergency nurse identified frequent users who were referred to family physicians. Three years later, in 2015, a part-time case manager was hired, but in a nearby clinic, not in the hospital. To improve coordination, two teams, each composed of a part-time dedicated nurse, a nursing assistant and a family assistant were created in two neighbouring localities near the hospital. However, the lack of support and unclear guidance from managers, and the demobilization of health and social providers due to the reorganization of healthcare system in 2015, led to the replacement of these teams by the part-time case manager from another area, which had to coordinate a much larger territory (see case F). Despite the many challenges this case manager faced (e.g. remote coordination, lack of time, creation of links with managers and health and social providers), and due to her expertise, this person was able effectively lead the CMP. The number of ED frequent users remained fairly stable over the study duration.

**E**	Liaison nurses assigned to this CMP included organizations that had pre-existing partnerships and a strong potential for collaboration in CMP, namely the hospital’s ED and mental health department, and the service area’s family medicine groups and local community health centre. In March 2015, just after its implementation, the CMP was interrupted due to the reorganization of the health and social services system. Thanks to the initiative of senior and intermediate managers, the CMP activities were temporarily resumed at the end of 2016, but stopped for a second time in the summer of 2017 due to persistent management and clinical staff’s instability. Given this short time frame (six months), the new nurse case manager was unable to create and consolidate collaboration among all CMP organizations. In March 2018, a new part-time social worker case manager was assigned to the CMP. In December 2018 (about the end of the qualitative data collection period), this case manager had still not received a training. The number of ED frequent users remained fairly stable for the duration of this study.

**F**	The CMP began in 2013, initially with a dyad composed of a nurse and a social worker. During the first year, human resources turnover in the hospital resulted in program interruptions. Following the reorganization of healthcare services in 2015, only the nurse stayed part-time as the case manager and focussed for the most part on elderly patients. The geographic proximity and leadership of this person helped to nurture partnerships with ED social workers, physicians and family medicine groups. For example, the case manager established a formal referral structure that linked family physicians, nurses, patients, and himself. By the end of the data collection, the case manager had finally been assigned full time to implement and execute the CMP in cases D, E and F. There were now enough human resources to deploy the CMP and consolidate existing partnerships. The number of ED frequent users decreased over the study period.


### Cross-case results

***Tables 3, 4, 5*** present the cross-case results. The outcomes (see the legend) are identified according to the five categories of Chaudoir et al. framework.

**Table 3:** Structural, environmental, and organizational characteristics influencing integrated care, self-management and health services use for each case.


CATEGORIES EMERGING CHARACTERISTICS	OUTCOMES					

	CASE A	CASE B	CASE C	CASE D	CASE E	CASE F

**Structural and environmental-level factor**						

(+) Proximity between providers		↑patient support (1.1)		↑ communications between healthcare professionals (1.2) ↑ healthcare transition (1.6)	↑ communications between healthcare professionals (1.2) ↑ healthcare transition (smaller ↑ for remote services) (1.6)	↑ collaboration with medical teams leading to ↑ comprehensive care and ↑ understanding of patient needs (1.1) ↑ communication between case manager, managers and healthcare professionals (1.2)

**Organizational-level factor**						

(–) Staff turnover and healthcare teams’ instability		↓continuity of care (1.6)		↓ case manager follow-up with healthcare professionals (1.2)	↓ continuity of care and ↑ repetition of patient medical history (1.6)	↓ consistency of patient support (1.1) ↓ patient information (1.2) ↓ continuity of care (1.6)

(+/–) Information access/No information access			↑response to patient needs (1.1) ↓ inadequate use of healthcare services given emergency nurses’ access to ISP* (3)	↓ assessment of patient situation (1.3) ↓ continuity of care between hospital and clinics (1.6) ↓ continuity of care when ISP not accessible to all healthcare professionals (1.6)	↑ response to patient needs (1.1) ↑ communication between case manager, ED physicians, and liaison and mental health nurses (1.2)	↑ health care transitions (1.6)

(+) Manager support		↑ service access for patients with most complex needs (1.1) ↑ service trajectories (1.6) ↓ use of health and social care services (3)				↑ case manager legitimacy and autonomy → new trajectories of care and ↑ continuity of care (1.6) ↓ use of healthcare services (3)


ISP: Individualized services plan.

**Table 4:** Practitioner and patient characteristics influencing integrated care, self-management and health services use for each case.


CATEGORIES EMERGING CHARACTERISTICS	OUTCOMES					

	CASE A	CASE B	CASE C	CASE D	CASE E	CASE F

**Practitioner-level factor**						

(+) Case manager leadership (skills, attitudes and personal qualities, previous experience, networking)		↑ access to other healthcare providers; leads to better follow-up and ↓ healthcare use (3)		↑ patient-centred care and ↑ access to adapted services (1.1) ↑ continuity of care (1.6) ↓ ED visits (because new CTs (3)		

(+) Provider engagement					↑ collaboration, communication and exchange of patient’s information between local community health centre and the hospital (1.2)	

(+) Inter-professional collaboration	↑ follow-up and a ↑ response to patient needs (1.1)		↑ sharing and discussion on patient information (1.2) ↓ healthcare use (3)		↑ access to clear and concise patient information (1.3) ↑ continuity of care and care planning (1.5, 1.6) ↑ consultations with other healthcare professional (patient doesn’t need to repeatedly see doctor for referrals) (3)	↑ knowledge of patient’s overall situation (1.1) ↑ communication between healthcare professionals (1.2) ↑ patient knowledge of care plan (1.3) ↓ patient repeating their medical history (1.6) ↑ patient appropriate use of resources (3)

**Patient-level factor**						

(+) Anxious patient	↑ patient confidence (given reassurance from case manager) (2) ↓ ED visits (3)					

(+) Patient with self-management skills		↑ patient proactivity in their health and healthy lifestyle choices (2) ↓ ED visits (3)		↑ patient proactivity in their care and healthy lifestyle choices (2) ↓ ED visits (3)		


CT: care trajectory; ED: emergency department.

**Table 5:** Characteristics of the innovation (the CMP) influencing integrated care, self-management and health services use for each case.


CATEGORIES EMERGING CHARACTERISTICS	OUTCOMES					

	CASE A	CASE B	CASE C	CASE D	CASE E	CASE F

**Innovation-level factor**						

(+) Individualized service plan (ISP)	↑ patient support and follow-up (1.1) ↑ patient involvement in their care (1.4) ↑ access to care (1.6)	↑ care planning (1.5)	↑ inter-professional communication (1.2)	↑ inter-professional collaboration, proximity and knowledge among healthcare professionals, which ↑ communication (1.2), information sharing and discussion (1.3) and healthcare transitions (1.6)	↑ healthcare professionals’ knowledge of the ISP (1.3)	

(+) Consideration of patient and family needs		↑ patient adherence to the program (1.1) ↓ healthcare use (3)		↑ case manager’s information access (1.3) ↑ patient participation in shared decision-making (1.4)	↑ patient-provider relationship of trust, thus ↑ response to patient needs (1.1) and self-management support (2)	↑ patient’s involvement in shared decision-making (1.4)

(+) Self-management support approach			↑ patient confidence, which leads to decreases their health services use (3)			↑ case manager and patient relationship of trust, thus ↑ communication (1.2)

(+/–) Relatives’ participation in decision-making/relatives support	↑ knowledge of patient needs and situation, thus ↑ patient follow-up (1.1) ↑ bidirectional information sharing (1.2, 1.3)		↑ knowledge of patient needs and situation (1.1) ↑ patient and relatives’ awareness of the care plan (1.3)		↑ patient adherence to ISP (1.5) ↓ hospitalization when relatives take patient to ED (3)	↑ response to patient needs (if very complex needs) (1.1) ↓ services use (3) ↑ use of care plan (1.5) by patients with low literacy (due to self-management support) (2)

(+/–) Case manager access to information			↑ relevance of response to patient needs (1.1) ↑ ISP efficiency (1.5)		↑ inter-professional communication (1.2)	↑ continuity of care (1.6)


ISP: Individualized services plan.

**Legend for *Tables 3, 4*** and ***5***

Outcomes associated with each CMP characteristic

**1** Integrated care
  **1.1** Consideration of patient and family needs
  **1.2** Communication with the patient and between practitioners
  **1.3** Access to information
  **1.4** Patient involvement in decision-making
  **1.5** Care planning
  **1.6** Transitions between various health professionals and practitioners
**2** Self-management
**3** Health services use

In the tables, the arrows represent an increase (↑), a decrease (↓), or an effect on another outcome (→), while the + and – signs represent contextual factors having a positive or negative impact on the implantation of CMPs.

### Cross-Case Synthesis

The skills, leadership and experience of the case manager seem to be the characteristics of the CMP that have the most positive influence on patient experience of integrated care, self-management and healthcare services use. The case manager’s leadership was critical in both successful cases (C and F), i.e. where we observed a decrease of ED visits. Their coordination, communication and networking skills improved integrated care by facilitating collaboration among professionals and also the transitions between health services, for which information access was a key. These improvements were also observed when the case manager was experienced, well-known in his/her workplace (C and F) and located near the providers (cases D, E, F).

Regarding the other characteristics of the CMP, four stand out from our cross-case analysis: 1) the individualized services plan (all cases), 2) patient and family needs assessment (all cases), 3) patient and family participation in decision-making (all cases), and 4) the self-management approach (cases C, D and F).

Our results suggest that where staff turnover and thus, health care team instability, was present due to organizational issues and the health system reorganization (cases B, D and E), negative impacts on care integration, especially regarding communication and care transitions, were observed. However, when case managers were well supported by their managers (cases B, E and F), they had the opportunity to create more personalized care trajectories. Therefore, patient transition through care pathways was optimized and their use of services was more appropriate. Reassurance of patients by their case manager appears to be particularly important for those with anxiety as it seems to have contributed to a reduction in their ED visits.

## Discussion

This study underscores the necessity of an experienced, knowledgeable, and well-trained case manager with strong interpersonal skills to optimize CMP implementation such that patients are more proactive in their care and their outcomes improve. These qualities improve care coordination which is one of the main components of CMP [30,31]. Similarly, Ross et al. pointed out that the case manager skills such as ability to develop good interpersonal relationships, problem-solving, negotiation and brokerage, prescribing qualifications play a key role to facilitate CMP implementation and improve outcomes [32]. Case manager training could include a focus on these skills. Indeed, a qualitative systematic review by Joo et al. revealed that insufficient training was a barrier to the case manager’s role [33]. Likewise, our results also underscored the importance of adequate training, but also that it can be challenging to ensure such training when there is a high turnover of case managers. Hong et al. provide a potential solution to this by suggesting that all care team members receive training, in order to build a relationship of trust with the patient [31].

To improve integrated care, although coordination by a skilled case manager is the core of case management, self-management support is important for CMP as a whole [14,34,35]. Self-management support seeks to improve patients’ knowledge and awareness of their care plan, self-efficacy, sense of control over their condition, and motivation to take more responsibility for their health [36,37]. To effectively provide this support, case managers should adopt an approach that is relevant, meaningful and centred on patient needs [32]. When the patient and caregiver manage the patient’s care adequately, their use of healthcare services is more appropriate and reduced rates of readmission are observed [38]. Furthermore, encouraging patients and their families to participate in decisions regarding the ISP better meets patient needs, promotes patient and family involvement in patient care and leads to fewer ED visits [35,39,40].

It could be argued that in-depth descriptions of the six CMP settings studied would be helpful to judge whether the results of this study are transferable to similar healthcare system settings [41]. However, given that the six CMP are heterogenous in terms of the populations they serve, their urban and rural environments, their size, the types of providers, among other key features (see ***Table 1***), this aspect increases the theoretical transferability of the results. That said, this study’s findings should be considered in light of some limitations that could be addressed in future research. First, only one source of quantitative data (ED visits) was used to measure CMP efficacy. Second, the qualitative data did not provide much insight into the factors linked to the ‘patient’ category of outcomes outlined in the Chaudoir et al. conceptual framework. Third, the qualitative results are relevant to many contextual factors in the other five categories of outcomes, but only those regarding the outcomes of interest (i.e., patient experience of integrated care and integrated care) are reported. Fourth, the case managers’ activities were not measured and evaluated. To further increase the credibility of the results, survey studies could be conducted with validated questionnaires that assess the impact of CMP on patients and the results could be triangulated with those presented herein. Finally, exploring system or organization level outcomes could complete the picture of the impact of CMP on frequent users’ health outcomes.

Studying CMP as they unfold is crucial to building the knowledge base regarding the components of CMP and the roll-out required to improve integrated care. This study is one of few that explore the implementation of CMP for frequent users of ED services in hospital settings. Additional implementation studies conducted in differing contexts or healthcare systems would be useful to confirm and further enrich the findings. In this regard, Malebranche et al. recently suggested that further research was needed to better understand the advantages and disadvantages of implementing case management as primary care program versus predominantly ED or hospital-based one [42]. Teper et al.’s systematic review of CMP implementation in primary care settings identified common facilitators and barriers of CMP implementation in hospital settings including case managers’ skills, training, and relationship building and team communication practices [43]. In a systematic mixed studies review on the barriers of CMP implementation for people with dementia in community-based primary health care, Khanassov et al. also reported the importance of communication between case managers and other professionals and services [44]. Identifying contextual barriers to CMP implementation can help to select more effective implementation strategies resulting in increased positive outcomes [44,45].

Based on the results of the study, recommendations can be made to senior and intermediate managers and clinicians for the planning and implementation of CMP. Senior managers should ensure ongoing support for the implementation of CMP and information sharing among health professionals. They should ensure stability in the health and social care teams, especially to maintain an experienced case manager. They also have a responsibility to promote the culture of a person-centred approach, i.e. one that encourages the consideration of patients’ needs and shared decision-making. Intermediate managers should facilitate the skills, leadership and experience of the case manager, as well as his/her proximity to providers. They will need to focus on the case manager’s skills during the hiring process and provide quality training in case management with frequent users. In addition, intermediate managers should foster professional development by, for example, allowing time for the case manager to participate in a community of practice or co-development activities. Clinicians must consider the needs of patients and their families when implementing the CMP. They should also provide support to patients and encourage their autonomy and involve them and their families in decision-making.

## Conclusion

This study underscores the necessity of an experienced, knowledgeable and well-trained case manager with interpersonal skills to optimize CMP implementation such that patients are more proactive in their care and their outcomes improve. Providers’ access to the individualized services plan, consideration of the needs of the patient and family members, their participation in decision-making, and the self-management approach, also impact patient experience of integrated care, self-management and services use.
